# Effects of Calcination Temperature on the Synthesis of One-Pot Sol-Gelled Barium Titanate Powder and Its Performance as an Endodontic Radiopacifier

**DOI:** 10.3390/ma17112701

**Published:** 2024-06-03

**Authors:** Pei-Jung Chang, May-Show Chen, Chi-Han Cheng, Yuh-Jing Chiou, Chin-Yi Chen, Cherng-Yuh Su, Chung-Kwei Lin

**Affiliations:** 1 Graduate Institute of Manufacturing Technology, National Taipei University of Technology, Taipei 106, Taiwan; t110569006@ntut.org.tw; 2 Research Center of Digital Oral Science and Technology, College of Oral Medicine, Taipei Medical University, Taipei 110, Taiwan; maychen@tmu.edu.tw (M.-S.C.); chiou@gm.ttu.edu.tw (Y.-J.C.); chencyi@fcu.edu.tw (C.-Y.C.); 3 School of Dentistry, College of Oral Medicine, Taipei Medical University, Taipei 110, Taiwan; 4 Division of Prosthodontics, Department of Dentistry, Taipei Medical University Hospital, Taipei 110, Taiwan; 5 School of Dental Technology, College of Oral Medicine, Taipei Medical University, Taipei 110, Taiwan; b210109019@tmu.edu.tw; 6 Department of Chemical Engineering and Biotechnology, Tatung University, Taipei 104, Taiwan; 7 Department of Materials Science and Engineering, Feng Chia University, Taichung 407, Taiwan; 8 Department of Mechanical Engineering, National Taipei University of Technology, Taipei 106, Taiwan

**Keywords:** sol-gel, barium titanate, mineral trioxide aggregates, radiopacity, diametral tensile strength, biocompatibility

## Abstract

Barium titanate (BaTiO_3_, BTO), conventionally used for dielectric and ferroelectric applications, has been assessed for biomedical applications, such as its utilization as a radiopacifier in mineral trioxide aggregates (MTA) for endodontic treatment. In the present study, BTO powders were prepared using the sol-gel process, followed by calcination at 400–1100 °C. The X-ray diffraction technique was then used to examine the as-prepared powders to elucidate the effect of calcination on the phase composition and crystalline size of BTO. Calcined BTO powders were then used as radiopacifiers for MTA. MTA-like cements were investigated to determine the optimal calcination temperature based on the radiopacity and diametral tensile strength (DTS). The experimental results showed that the formation of BTO phase was observed after calcination at temperatures of 600 °C and above. The calcined powders were a mixture of BaTiO_3_ phase with residual BaCO_3_ and/or Ba_2_TiO_4_ phases. The performance of MTA-like cements with BTO addition increased with increasing calcination temperature up to 1000 °C. The radiopacity, however, decreased after 7 days of simulated oral environmental storage, whereas an increase in DTS was observed. Optimal MTA-like cement was obtained by adding 40 wt.% 1000 °C-calcined BTO powder, with its resulting radiopacity and DTS at 4.83 ± 0.61 mmAl and 2.86 ± 0.33 MPa, respectively. After 7 days, the radiopacity decreased slightly to 4.69 ± 0.51 mmAl, accompanied by an increase in DTS to 3.13 ± 0.70 MPa. The optimal cement was biocompatible and verified using MG 63 and L929 cell lines, which exhibited cell viability higher than 95%.

## 1. Introduction

Barium titanate (BaTiO_3_, BTO), a typical perovskite-structured electronic material for dielectric and ferroelectric applications [1,2,3], has been used in piezoelectric transducers, actuators and sensors, multilayer ceramic capacitors, ultrasonic and electro-optical devices, thin films for photonics, thermistors, etc. The perovskite structure of BTO allows it to induce charges during deformation. The deformation of BTO causes an asymmetric shift of dipoles in the crystal lattice and generates piezoelectricity. This makes BTO an attractive biomaterial for biomedical applications in the human body where bioelectricity plays an important role, such as in the neuronal system [4], cardiology [5], and bone regeneration [6].

Conventionally, solid-state reaction and heat treatment at relatively high temperatures are used to synthesize BTO [7,8]. Similar to the solid-state reaction, BTO powder can be obtained using a high-energy ball milling process, in which starting powder mixtures of BaCO_3_ and TiO_2_ are ball-milled for a period of time to refine the starting powders and undergo heat treatment at high temperature [9]. When using BaO and TiO_2_ as starting materials, a mechanochemical reaction may occur due to the high energy input during ball milling, directly resulting in the formation of BTO powder [10]. The above-mentioned solid-state methods, however, may suffer contamination from grinding agents and inhomogeneity with a relatively wide grain size distribution. Alternative methods involving chemical means, such as the solvothermal method [11], hydrothermal synthesis [12], coprecipitation [13], and the sol-gel process [14,15], have been used to prepare BTO powders created from various barium and titanium cation precursor solutions. BTO powders with or without modification (adding a ternary cation, surfactant, etc.) can be synthesized through different reaction mechanisms and controlled reactor environments. The resulting BTO powder may exhibit different crystalline structures, sizes, shapes, particle size distributions, etc., and be used in a wide variety of applications [16,17].

Biomedical applications of BTO materials have been widely investigated and recently reviewed by Sood et al. [18], covering drug/gene delivery, cancer therapy, bioimaging, tissue engineering, wound healing, biosensors, wearable and implanted bioelectronic devices, etc. In dental applications, PMMA dentures with 40 wt.% barium titanate addition exhibited antifungal effects due to the piezoelectric charges induced by BTO [19]. Choi et al. reported that a 40–60 wt.% BTO-added tricalcium silicate exhibited proper radiopacity, favorable biocompatibility, and beneficial bone regeneration effects [20]. In the previous investigation, we demonstrated that BTO prepared using a combination of heat treatment and high-energy ball milling could be potentially used as a radiopacifier for mineral trioxide aggregates (MTAs). BTO-13 (mechanical milling for 3 h and heat treatment at 1300 °C for 2 h) was optimal and MTA-like cement mixed with 30% BTO-13 and solidified using 10% CaCl_2_ solution exhibited the best performance for potential clinical application [21].

MTA typically consists of 75% Portland cement, 25% bismuth oxide, and 5% gypsum. It has been widely used in endodontic treatment for repairing lateral perforations, apexification, direct pulp capping, and root end filling [22,23]. During the clinical application of MTA, the hydration of Portland cement grants it sealing ability and mechanical strength, whereas bismuth oxide provides the required radiopacity to show the effects of endodontic treatment. Conventional MTA, however, may experience difficulties with manipulation and long setting times during preparation. Mechanical strength and intrinsic cytotoxicity are additional concerns after solidification, whereas tooth discoloration becomes an important issue after endodontic therapy. MTA-like cements with adjusted constitutions, alternative radiopacifiers, and various solidifying solutions have been investigated to address these issues. For instance, Sen et al. [24] reported the radiopacity performance of various commercially available calcium silicate cements. Among them, ProRoot MTA with Bi_2_O_3_ as radiopacifier exhibited the highest radiopacity of 4.32 ± 0.17 mmAl, whereas Biodentine using ZrO_2_ was the lowest (2.29 ± 0.21 mmAl). In addition to radiopacity, Camilleri et al. [25] investigated the hydration and bioactivity of selected calcium silicate cements. Cytoxicity and tooth discoloration were studied by Oliveira et al. [26] and Lee et al. [27]. Additional health concerns include bismuth oxide-induced tooth discoloration and the release of bismuth into adjacent tissues, blood, and organs [28].

MTA-like cements made with a wide variety of materials have been investigated. The processing techniques, however, are seldom explored. Materials prepared through physical or chemical routes may exhibit different materials characteristics (e.g., particle size, shape, distribution, and impurities) and affect clinical performance [29,30]. The one-pot sol-gel process can simplify the sol-gel process and synthesize unique powders for various applications [31,32,33]. In the present study, BTO powder was prepared using the one-pot sol-gel process and calcination. The effect of calcination temperature on radiopacity and diametral tensile strength after 1 and 7 days of solidification was investigated. The biocompatibility of optimal BTO-added MTA-like cement was also evaluated using MG63 and L929 cell lines to confirm its feasibility for practical application.

## 2. Materials and Methods

### 2.1. One-Pot Sol-Gelled Barium Titanate Synthesis, Calcination, and Characterization

One-pot sol-gel process followed by calcination was used to prepare BTO powders. Barium hydroxide octahydrate (Ba(OH)_2_·8H_2_O, Fluka, ≥98%) and titanium(IV) n-butoxide (Ti(O(CH_2_)_3_CH_3_)_4_, Alfa Aesar, 99+%) were used as precursors for the preparation of barium titanate powder. The synthesis procedures are described as follows: 12.9 g of barium hydroxide octahydrate and 13.6 g of titanium (IV) n-butoxide were dissolved in 200 mL deionized water and stirred for 2 h at 60 °C. Ammonium hydroxide (NH_4_OH, J.T. Baker, 28–30%) was subsequently added to the precursor solution and aged for 3 h. After aging, the solution was dried in a glass culture dish for 16 h at 80 °C. The dried powder was then calcined at various temperatures (400, 600, 800, 900, 1000, 1100 °C) for 1 h.

A thermogravimetric analyzer TGA-2, Mettler-Toledo, Greifensee, Switzerland) was used to examine the thermogravimetry analysis (TGA), differential scanning calorimetry (DSC), and derivative thermogravimetry (DTG) of dried powder before calcination. An amount of 20 mg of powder was analyzed within 25 °C to 1000 °C at a 10 °C/min heating rate under an ambient air environment. A D2 PHASER X-ray diffractometer (Bruker, Billerica, MA, USA) was used to analyze the crystalline structure of calcined powders. Cu Kα radiation (λ = 1.542 Å) was generated using a voltage of 30 kV and a current of 10 mA, filtered by nickel film, and used for examination. In addition, the powder’s morphology was observed using a field emission scanning electron microscope (SU-8200, Hitachi, Tokyo, Japan).

### 2.2. Preparation and Evaluation of BTO-Added MTA-like Cements

Portland cement (Gold Star Co., LTD, New Taipei City, Taiwan), calcined barium titanate powder (20 and 40 wt.%), and deionized water (Portland cement/water = 3) were mixed to prepare MTA-like cements. The powder mixture was first blended by a homogenizer (Prep-CB6, Medclub Scientific Co., Ltd., Taoyuan, Taiwan) for 10 min. Subsequently, deionized water was added to the blended powder and mixed further using a Vortex-Genie 2 mixer (Scientific Industries, Inc., Bohemia, NY, USA) for 15 s. The paste was then filled into acrylic molds with various dimensions depending on the following experiments. After filling into the molds, the MTA-like cement was placed for 1 day and 7 days in an incubator that was kept at 37 °C with a humidity of ~90% to simulate the oral environment of the human body.

For radiopacity measurements, disc samples (10 mm diameter and 1 mm thickness) were prepared and examined using a VX-65 dental X-ray system (Vatech Co., Ltd., Yongin Si, Gyeonggi-Do, Republic of Korea). Six samples (N = 6) and a referenced step-wedge aluminum block were exposed simultaneously with an occlusal radiographic film (Imaging plate size 2; Dürr Dental, Bietigheim-Bissingen, Germany) at a focus-film distance of 30 cm [34]. Imaging processing software (Image J 1.52a, Wayne Rasband, National Institutes of Health, Bethesda, MD, USA) was used to analyze the image and determine the corresponding radiopacity. In addition, cylindrical samples (6 mm diameter and 5 mm height; N = 6) were examined using a texture analyzer machine (TA. XT plus, Stable Micro Systems, Godalming, UK) at a test speed of 6.00 mm/min to determine the DTS value equaling 2*F*/π*bw*, where *F* is the maximum applied load (N), *b* is the diameter (mm), and *w* is the height (mm) of the cylinder.

### 2.3. In Vitro Biocompatibility of Optimal BTO-Added Cements

The biocompatibility of optimal BTO-added MTA-like cement was tested using disc samples (10 mm diameter and 1 mm thickness, same as those for radiopacity experiments). Based on the radiopacity and DTS evaluation, the in vitro biocompatibility of optimal MTA-like cement was investigated by using Alamar Blue Cell Viability Reagent with human osteoblast-like osteosarcoma MG-63 and murine normal fibroblast L929 cells [35]. Before seeding, both cells were cultured in a minimal essential medium (MEM, Gibco, Thermo Fisher Scientific Inc., Waltham, MA, USA), supplemented with 10% fetal bovine serum (FBS, Sigma-Aldrich, Merck, Burlington, MA, USA) and 1% penicillin/streptomycin (PS, Gibco), and incubated at 37 °C under 5% CO_2_ environment. Seeding of MG63 and L929 cells was performed in a 96-well plate with a density of 10^4^ per well. Next, the cells were cultured in extracts, with concentrations of 1/10,000, 1/1000, or 1/100, of the optimal MTA-like cement soaked for 24 h. Each examining condition was performed with four replicates and both cells without extracts served as the control group.

Cell proliferation was evaluated using Alamar Blue (Thermo Fisher Scientific, Waltham, MA, USA). After culturing for 1 day, media from the 96-well plate were replaced by a fresh culture medium with 10 μm/mL of Alamar Blue solution and incubated for 4 h at 37 °C under a 5% CO_2_ environment. Afterwards, a microplate photometer (The SpectraMax^®^ iD3, Molecular Devices, San Jose, CA, USA) was used to measure the fluorescence at a wavelength of 530–590 nm to calculate the cell viability [36]. Furthermore, a ZEISS AXIOVERT 200 inverted phase-contrast microscope (ZEISS, Oberkochen, Germany) was used to observe the cell morphology.

### 2.4. Statistical Analysis

For radiopacity and diametral tensile strength, statistical analysis was performed using SPSS software (version 18.0, IBM Corporation, New York, NY, USA) with Student’s *t*-test at various confidence intervals of 0.05, 0.01, and 0.001, respectively.

## 3. Results and Discussion

### 3.1. Characterization of Sol-Gelled Barium Titanate Powders

**Figure 1:**
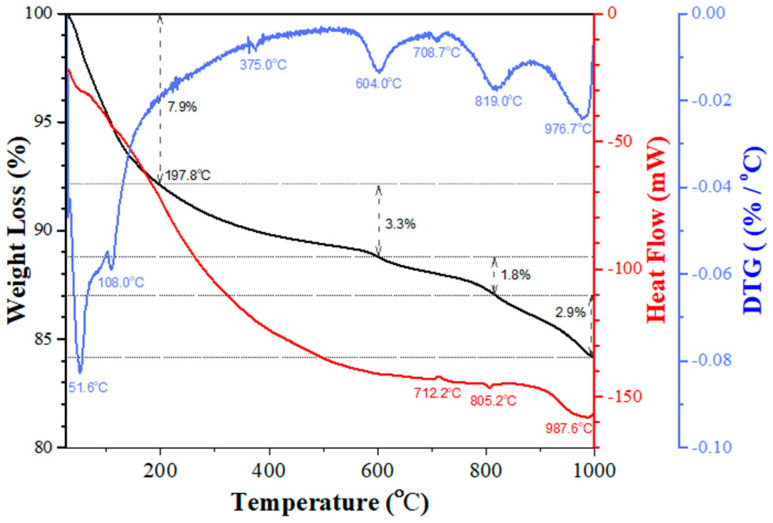
Thermal analysis of sol-gelled BTO powder.

The sol-gelled product before calcination exhibited an amorphous phase without any crystalline phases. The dried sol-gelled powder was examined using thermal analysis and Figure 1 shows the corresponding TGA/DSC/DTG curves. It can be noted that the TGA curve (the black line in Figure 1) shows a descending trend within the testing temperature range from room temperature to 1000 °C. A rapid weight loss (~7.9%) was observed at the beginning of the thermal analysis from room temperature to ~200 °C. This can be attributed to the water evaporation and burnout of some organic solvents. The TGA curve exhibited a relatively smooth weight loss of ~3.3% within the temperature range from 200 to 600 °C. After reaching 820 °C, the speed of weight loss increased slightly and ~1.8% weight loss was observed. Towards the end of the thermal analysis (from 820–1000 °C), the rate of weight loss increased again to ~2.9%. The DSC curve (the red line) exhibited a similar trend to the TGA curve, whereas the DTG curve (the differentiation of the TGA curve, the blue line) revealed some interesting findings. A sharp endothermic peak at 80 °C corresponding to water evaporation was observed. Three broad peaks were exhibited, respectively, at relatively high temperatures of 600, 820, and 980 °C. The first two endothermic peaks can be attributed to the formation of BaCO_3_ and BaTiO_3_, whereas the last one was due to the transition of Ba_2_TiO_4_ to BaTiO_3_. This shows a similar result to one reported in the literature [37]. The thermal analysis results and possible reactions are summarized in Table 1.

**Figure 2:**
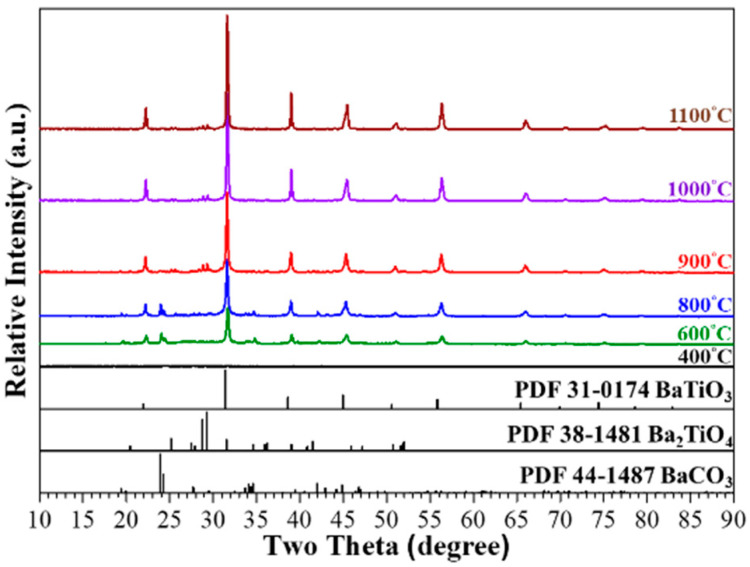
XRD patterns of sol-gelled BTO powder after calcination at different temperatures for 1 h. The bottom three patterns are referenced powder diffraction files (PDF) from International Centre for Diffraction Data (ICDD).

**Figure 3:**
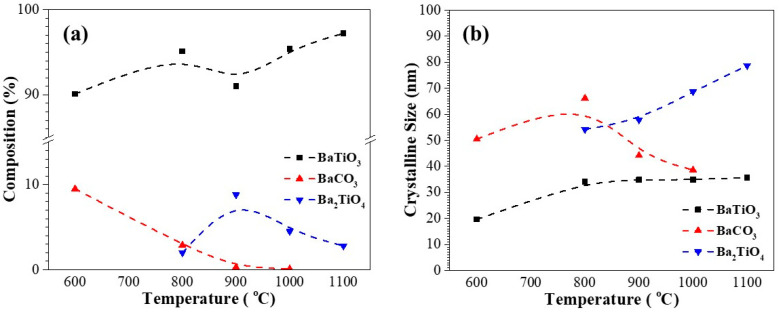
(**a**) Compositions and (**b**) crystalline sizes of sol-gelled BTO powder after calcination at different temperatures for 1 h.

Based on the thermal analysis results, the calcination of sol-gelled powder was performed at 400, 600, 800, 900, 1000, and 1100 °C for 1 h, respectively. Figure 2 shows the corresponding XRD curves. No crystalline peaks can be observed for 400 °C-calcined powder, indicating an amorphous phase. Major BaTiO_3_ (ICDD PDF card no. 31-0174) and minor BaCO_3_ (ICDD PDF card no. 44-1487) phases were exhibited after calcination at 600 °C for 1 h. Further increasing the calcination temperature to 800 °C resulted in similar XRD patterns. However, the peak intensities of BaTiO_3_ increased slightly and indicated an increase in the crystallinity and crystalline size of the BTO powder. In addition, some small peaks belonging to Ba_2_TiO_4_ (ICDD PDF card no. 38-1481) were observed in the 800 °C-calcined powder [38]. After calcination at 900 °C, the diffraction peaks of BaCO_3_ became ambiguous, and those of BaTiO_3_ and Ba_2_TiO_4_ grew continuously. After further increasing the calcination to 1000 and 1100 °C, BaCO_3_ almost disappeared and was accompanied by a continuous decrease in Ba_2_TiO_4_ and increase in BaTiO_3_. In order to further reveal the phase evolution during various calcination stages, the XRD patterns were analyzed using Rietveld’s fitting method and Figure 3 shows the corresponding results. As shown in Figure 3a, the 600 °C-calcined powder consisted of BaTiO_3_ (90.1%) and BaCO_3_ (9.9%). The percentage of BaCO_3_ decreased continuously with increasing calcination temperature and the percentages were 2.9, 0.3, and 0.0 for 800, 900, and 1000 °C, respectively. Formation of Ba_2_TiO_4_ was observed after calcination at 800 °C. The percentage was 2.0% at 800 °C, which increased to a maximum of 8.8% at 900 °C, decreased continuously thereafter, and was 2.8% at 1100 °C. In contrast to that of Ba_2_TiO_4_, the percentage of BaTiO_3_ increased from 90.1% (600 °C) to 95.1% (800 °C), decreased to 91.0% at 900 °C, and increased continuously to the end of calcination. The constitution of 1100 °C-sol-gelled powder was BaTiO_3_ (97.2%) with some residual Ba_2_TiO_4_ phase (2.8%). Using Rietveld’s fitting method, the crystalline sizes of various phases were also estimated by using Scherrer’s equation with a shape factor of 0.9. Figure 3b shows the variation in crystalline size for BaTiO_3_, BaCO_3_, and Ba_2_TiO_4_ as a function of calcination temperature. It can be noted that the crystalline sizes of BaTiO_3_ and Ba_2_TiO_4_ increased continuously with increasing calcination temperature and Ba_2_TiO_4_ exhibited a larger crystalline size than BaTiO_3_. The crystalline size for BaTiO_3_ increased from 19.63 nm (600 °C) to 35.66 nm (1100 °C) and that of Ba_2_TiO_4_ was 54.19 nm and 78.73 nm for 800 and 1100 °C, respectively. The crystalline size of BaCO_3_, however, was 50.48 nm at 600 °C, reached a maximum (66.21 nm) at 800 °C, and decreased gradually to 38.62 nm at 1000 °C. Table 2 summarizes all the crystalline phases and corresponding crystalline sizes for the calcined sol-gelled powders.

**Figure 4:**
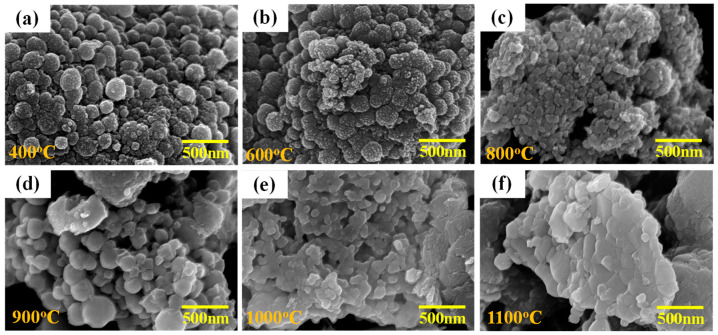
SEM images of sol-gelled BTO powder after calcination at (**a**) 400, (**b**) 600, (**c**) 800, (**d**) 900, (**e**) 1000, and (**f**) 1100 °C for 1 h.

Figure 4 shows the SEM images of the corresponding calcined sol-gelled powder. It can be noted that, at a relatively low calcination temperature of 400 °C (Figure 4a), the sol-gelled powder exhibited a typical agglomeration phenomenon where individual particles could be observed distinctly. Though a slight connection among individual particles was observed, a similar powder morphology (Figure 4b) was exhibited after calcination at 600 °C. The grain growth and sintering phenomena were more evident after calcination at 800 °C. As shown in Figure 4c, the boundaries among particles became ambiguous, with some large pores within agglomerated large particles. Further increasing the temperature to 900 °C (Figure 4d) resulted in large particles that grew continuously from the consumption of smaller ones. Compared to those in Figure 4a, relatively small separated particles with more pores (compared to Figure 4c) were observed. Interconnection among particles became more distinct, whereas individual particles were still visible together with a decrease in porosity after calcination at 1000 °C (Figure 4e). The sintering phenomenon became dominant when the calcination temperature was 1100 °C. As shown in Figure 4f, the boundaries among particles diminished and the porosity reduced significantly.

### 3.2. Performance of MTA-like Cements Prepared Using Sol-Gelled BaTiO_3_

As demonstrated above, except for the amorphous 400 °C-calcined powder, the calcined sol-gelled powder (600–1100 °C) exhibited a major BaTiO_3_ phase with minor BaCO_3_ and/or Ba_2_TiO_4_ phases. These powders were used as the radiopacifiers to prepare MTA-like cements for radiopacity and diametral tensile strength evaluations.

**Figure 5:**
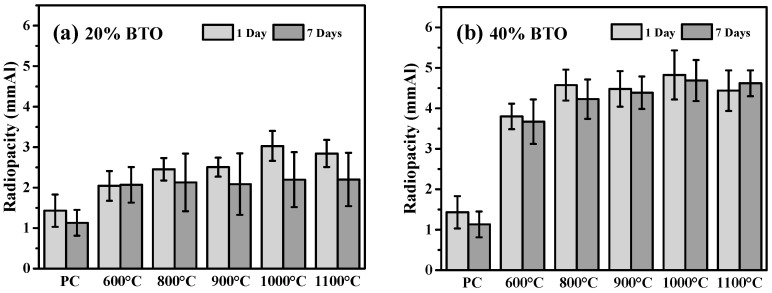
Radiopacity performance of MTA-like cements prepared by adding (**a**) 20 and (**b**) 40 weight percentages of sol-gelled powder.

**Figure 6:**
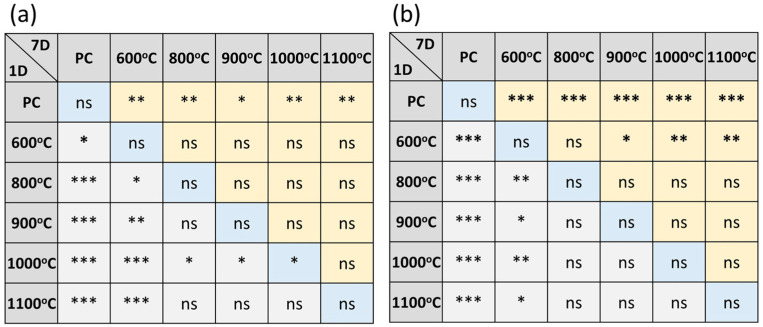
Statistical analysis of radiopacity performance for MTA-like cements prepared with (**a**) 20 and (**b**) 40 weight percentages of sol-gelled powder. “ns” designates no significant difference, whereas *, **, and *** indicate that these two sets of samples were statistically different at 95%, 99%, and 99.9% confidence intervals, respectively. The middle ones compare the same sets of samples after 1 day and 7 days of setting. Those in the lower triangle compare different samples after 1 day of setting, whereas those in the upper triangle compare different samples after 7 days of setting.

Figure 5 shows the radiopacities of MTA-like cements prepared by adding 20 wt.% and 40 wt.% calcined sol-gelled BTO powder. Without addition of the BTO radiopacifier, as shown in Figure 5a, PC exhibited a radiopacity of 1.43 ± 0.40 mmAl after 1 day of simulated environmental storage as suggested by the ISO standard [39]. This increased to 2.45 ± 0.28, 2.51 ± 0.23, 3.03 ± 0.37, and 2.84 ± 0.33 mmAl for MTA-like cements prepared using 800, 900, 1000, and 1100 °C-calcined BTO powders, respectively. In general, the radiopacity performance increased with increased calcination temperature. It reached a maximum of 3.03 mmAl for 1000 °C-calcined powder. After 7 days of simulated storage, a decrease in radiopacity for all the examined MTA-like cements was observed. The 1000 °C-calcined BTO still had the highest radiopacity of 2.20 ± 0.68 mmAl, but it did not meet the 3 mmAl required ISO standard [39]. This shows a similar trend to that reported in the literature, where a radiopacifier with a relatively low atomic number was used. Since the radiopacity increased with the increasing amount of radiopacifier, Figure 5b shows the corresponding results where a significant increase in radiopacity for all the MTA-like cements with 40 wt.% BTO addition was observed and all of them were higher than the requirement. Compared to those with 20 wt.% BTO addition, a similar tendency can be observed for 40 wt.% BTO addition. The 1000 °C-calcined BTO exhibited the highest radiopacities of 4.83 ± 0.61 and 4.69 ± 0.51 mmAl after 1 and 7 days of simulated storage, respectively. In addition, except for the 1100 °C-calcined BTO powder, a relatively lower radiopacity was exhibited after 7 days. The optimal MTA-like cements with 40% 1100 °C-calcined BTO powder exhibited a radiopacity of 4.83 mmAl (4.69 mmAl after 7 days of setting), which was higher than the human dentine (1.70 mmAl) and bovine mandibular cortical bone (3.43 mmAl) [40]. It is also comparable to those of Bi_2_O_3_-added ProRoot MTA [24] and MTA-like cements [41,42]. In practical clinical applications, though the optimal radiopacity met the ISO requirement (3 mmAl), it could still be responsible for low-detectable periapical extrusions. MTA-like cement with higher radiopacity could be useful in the presence of dynamic periapical resorption when using premixed CaSi sealers for either warm or cold obturation techniques for endodontic therapy. This can be achieved by increasing the amount of radiopacifier addition. Table 3 summarizes all the radiopacities for the MTA-like cements investigated in the present study. Detailed statistical analysis concerning the radiopacity performance in various conditions (samples after 1 day and 7 days of setting, and different sets of samples after 1 day and 7 days of setting, respectively) were performed and the results are shown in Figure 6.

**Figure 7:**
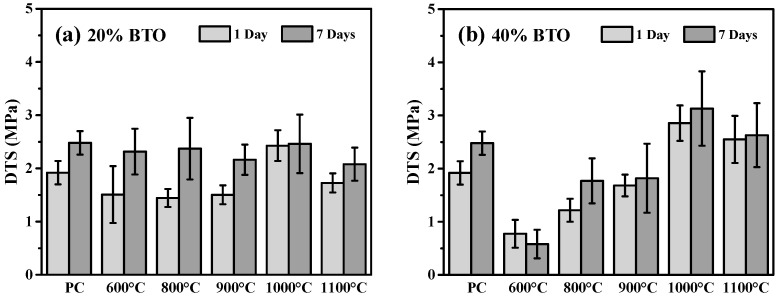
DTS of MTA-like cements prepared with (**a**) 20 and (**b**) 40 weight percentages of sol-gelled powder.

The mechanical properties of these MTA-like cements were evaluated by diametral tensile strength (DTS), and Figure 7 shows the corresponding results for those investigated in Figure 5. Generally, MTA-like cement with BTO addition exhibited a relatively lower DTS than pure Portland cement (1.92 ± 0.22 MPa). As shown by the light-grey bars in Figure 7a, the DTS was 1.51 ± 0.53, 1.44 ± 0.17, and 1.50 ± 0.18 MPa with 20 wt.% BTO powder calcined at 600, 800, and 900 °C, respectively. However, it is interesting to note that the DTS increased significantly to 2.43 ± 0.29 MPa using the 1000 °C-calcined BTO powder and then decreased to 1.73 ± 0.18 MPa with the 1100 °C BTO powder. After 7 days of storage, as shown by the dark-grey bars in Figure 7a, an improvement in DTS compared to their corresponding counterpart (DTS of MTA after 1 day of storage, the light-grey bars in Figure 7a) can be observed. The DTS for Portland cement was 2.48 ± 0.22 MPa and 2.32 ± 0.42, 2.37 ± 0.58, 2.16 ± 0.28, 2.46 ± 0.55, and 2.08 ± 0.31 MPa with BTO powder calcined at 600, 800, 900, 1000, and 1100 °C, respectively. Though all the MTA-like cement exhibited a slightly lower DTS value than pure Portland cement, no significant difference can be seen after 7 days of simulated storage.

**Figure 8:**
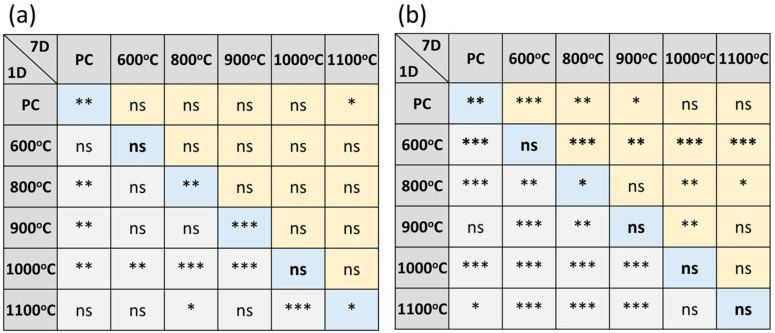
Statistical analysis of DTS performance for MTA-like cements prepared with (**a**) 20 and (**b**) 40 weight percentages of sol-gelled powder. “ns” designates no significant difference, whereas *, **, and *** indicate that these two sets of samples were statistically different at a 95%, 99%, and 99.9% confidence interval, respectively. The middle ones compare the same sets of samples after 1 day and 7 days of setting. Those in the lower triangle compare different samples after 1 day of setting, whereas those in the upper triangle compare different samples after 7 days of setting.

A relatively large variation in DTS, however, was observed for MTA-like cements prepared with the addition of 40 wt.% calcined BTO powders, Figure 7b. With 40 wt.% 600 °C-calcined BTO powder, the DTS (0.77 ± 0.26 MPa) was very low. The DTS increased with calcination temperature, reaching a maximum at 1000 °C and decreasing slightly with 1100 °C-calcined powder. The DTS was 1.22 ± 0.22, 1.68 ± 0.20, 2.86 ± 0.33, and 2.55 ± 0.44 MPa for 800, 900, 1000, and 1100 °C-calcined powders, respectively. After 7 days of simulated oral environmental storage, the DTS (0.58 ± 0.27 MPa) for 600 °C-calcined powder was even lower than the initial one, whereas the others showed a similar increasing trend to those prepared using 20 wt.% calcined powder. For MTA prepared with 40 wt.% 800, 900, 1000, and 1100 °C-calcined powders, the DTSs were 1.77 ± 0.42, 1.82 ± 0.65, 3.13 ± 0.70, and 2.63 ± 0.60 MPa, respectively. It should be pointed out that MTA-like cement prepared by using BTO powder calcined at relatively low temperatures (for instance, 600 °C) resulted in a sandy type of paste after mixing with solution, and possessed a relatively low DTS value. The 1000 °C-calcined powder was optimal and exhibited a suitable grain size for preparing MTA-like cement, and the DTS was the highest in the present study. All DTS values for the MTA-like cements are summarized in Table 4. Figure 8 shows the detailed statistical analysis concerning the DTS performance in various conditions. It can be noted that the MTA-like cement with 40 wt.% 1000 °C-calcined BTO powder addition exhibited a DTS similar to that of PC and was significantly higher than the others.

### 3.3. Biocompatibility Evaluation of Optimal MTA-like Cement

**Figure 9:**
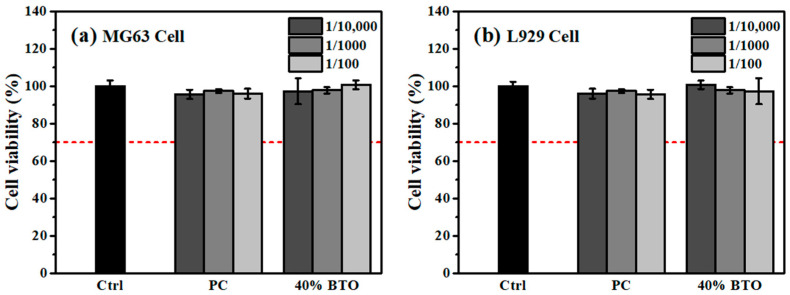
Cell viability of (**a**) MG63 and (**b**) L929 cells tested at different concentrations of extract from PC and 40 wt.% BTO. The cell viability was determined using Alamar Blue.

As shown by the resulting radiopacity and DTS, the MTA-like cement prepared using 1000 °C-calcined powder exhibited optimal performance and was investigated further concerning its biocompatibility with MG63 and L929 cell lines using the CCK8 kit. MG63 cells are similar to human osteoblasts and are considered reliable for testing biocompatibility [43], whereas L929 cells are more sensitive, and their use is recommended by the ISO standard [44]. Both MG63 and L929 cells were treated with three different concentrations of extracts (1/10,000, 1/1000, and 1/100) from MTA-like cement. According to the literature, they exhibit better biocompatibility at lower concentrations [45], whereas higher concentrations can be used to determine the toxicity exposure tolerance [46]. Figure 9a shows the cell viability results for MG63 cells. The MTA-like cement without radiopacifier (PC) and those prepared by adding 20 or 40 wt.% 1000 °C-calcined BTO powder exhibited cell viability higher than 95% when tested with three different levels of extract concentrations. Comparisons between the various MTA-like cements and the control group (100 ± 3%) showed no statistical differences. A similar trend can be observed when treated with ISO standard L929 cells, and Figure 9b shows the corresponding results. Compared with the control group (100 ± 3%), all MTA-like cements with or without radiopacifier tested in various extract concentrations exhibited cell viabilities higher than 95%. Table 5 lists all the biocompatibility results investigated in the present study.

**Figure 10:**
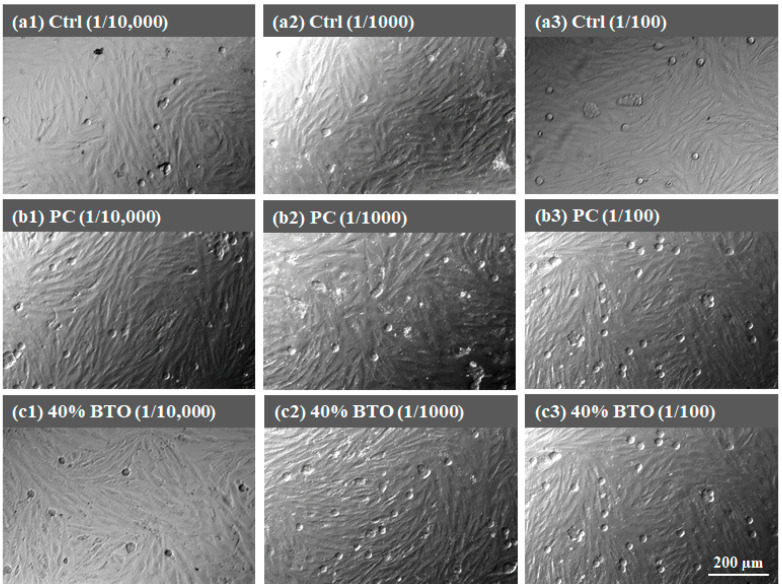
MG63 cell morphologies examined at different concentrations of extract from (**a1**–**a3**) control, (**b1**–**b3**) PC, and (**c1**–**c3**) 40 wt.% BTO.

**Figure 11:**
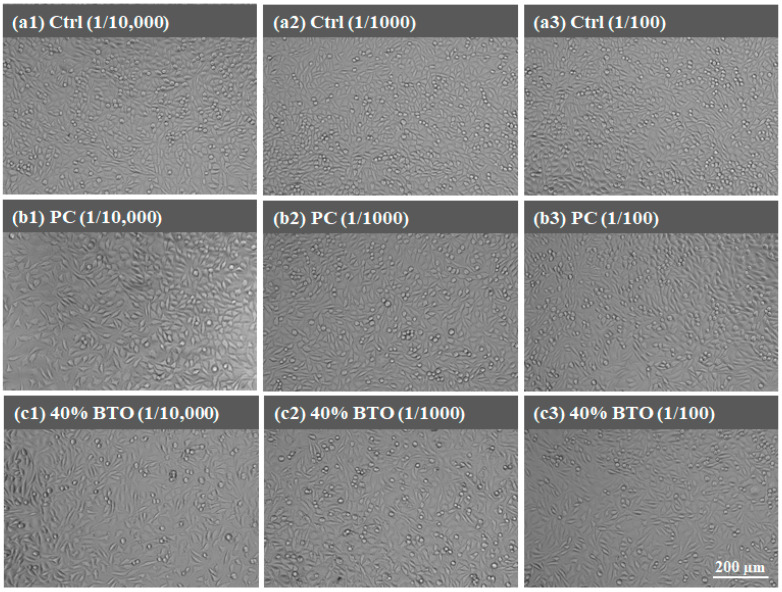
L929 cell morphologies examined at different concentrations of extract from (**a1**–**a3**) control, (**b1**–**b3**) PC, and (**c1**–**c3**) 40 wt.% BTO.

There is no doubt that all the MTA-like cements exhibited superior biocompatibility, with cell viability significantly higher than the 70% required by the ISO 10993-5 standard [47]. In order to further confirm the biocompatibility, the morphologies of MG63 and L929 cells were examined and Figure 10 and Figure 11 show the corresponding images for those investigated in Figure 9. Figure 10 shows the morphology of MG63 cells when tested with the extracts of varying concentrations from all MTA-like cement. A similar cell morphology compared to the control group was observed and MG 63 cells exhibited a typical fibroblast shape without any damage. Similar behavior can be observed when tested with L929 cells, for which all the cell appearances were similar to the control group. As shown in Figure 11, all L929 cells from various samples exhibited typical spindle-like fibers. This shows a similar result to reported in the literature [48].

It can be concluded that the MTA-like cement prepared using 40 wt.% 1000 °C-calcined BTO powder exhibited the best radiopacity and DTS performance and was biocompatible. It can be potentially used as a novel MTA material for clinical applications.

## 4. Conclusions

Barium titanate powders were prepared successfully using the sol-gel process followed by calcination at a temperature ranging from 600 to 1100 °C. The so-obtained powders exhibited a mixture of major BaTiO_3_ phase and minor Ba_2_TiO_4_ or BaCO_3_ phases. The calcined sol-gelled powders were then used as a radiopacifier to prepare MTA-like cements. The radiopacity and DTS performances of MTA-like cements generally increased with increasing calcination temperature and reached their maximum at 1000 °C. After storing in a simulated oral environment for 7 days, a decrease in radiopacity and an increase in DTS could be observed. By adding 40 wt.% 1000 °C-calcined BTO powder, the MTA-like cement exhibited optimal properties with a radiopacity of 4.83 ± 0.61 mmAl and a DTS of 2.86 ± 0.33 MPa. The radiopacity decreased to 4.69 ± 0.51 mmAl and the DTS increased to 3.13 ± 0.70 MPa after 7 days. The biocompatibility of the optimal cement was confirmed using MG 63 and L929 cell lines. Not only were the cell viabilities from various concentrations of extracts (1/100, 1/1000, and 1/10,000) better than 95%, but the cells also exhibited a similar morphology to those of control groups. In the present work, we have demonstrated that MTA-like cement with 40 wt.% 1000 °C-calcined BTO powder addition was biocompatible, exhibited the best performance, and possessed potential for endodontic applications.

## Figures and Tables

**Table 1:** Summary of thermal analysis results of sol-gelled BTO powder.

T_range_ (°C)	Wt. Loss (%)	DSC (°C)	DTG (°C)	Possible Reactions
RT-200	7.9	--	--	water evaporation and organic solvent burnout
200–600	3.3	--	--	BaTiO_3_ and BaCO_3_ formation
600–820	1.8	712.2 endo. 805.2 exo.	604.0 708.7 819.0	BaCO_3_ decomposition and Ba_2_TiO_4_ formation
820–1000	2.9	987.6 endo.	976.7	BaCO_3_ decomposition and transition of Ba_2_TiO_4_ to BaTiO_3_

**Table 2:** Crystalline phases and sizes for sol-gelled powders after calcination at various temperatures.

Calcination Temperature (°C)	Composition (%)			Grain Size (nm)		
	BaTiO_3_	BaCO_3_	Ba_2_TiO_4_	BaTiO_3_	BaCO_3_	Ba_2_TiO_4_
600	90.5	9.5	-	19.63	50.48	-
800	95.1	2.9	2.0	34.11	66.21	54.19
900	91.0	0.3	8.8	34.83	44.20	57.89
1000	95.4	0.1	4.5	34.92	38.62	68.73
1100	97.2	-	2.8	35.66	-	78.73

**Table 3:** Radiopacity (after 1 and 7 days of setting) of MTA-like cements prepared by adding 20 and 40 wt.% of various calcined BTO powders.

Radiopacity (mmAl)		PC	600 °C	800 °C	900 °C	1000 °C	1100 °C
20% BTO	1 Day	1.43 ± 0.40	2.04 ± 0.27	2.45 ± 0.28	2.51 ± 0.23	3.03 ± 0.37	2.84 ± 0.33
	7 Days	1.13 ± 0.32	2.06 ± 0.44	2.13 ± 0.71	2.09 ± 0.76	2.20 ± 0.68	2.20 ± 0.66
40% BTO	1 Day	1.43 ± 0.40	3.80 ± 0.31	4.57 ± 0.38	4.48 ± 0.44	4.83 ± 0.61	4.44 ± 0.50
	7 Days	1.13 ± 0.32	3.67 ± 0.55	4.23 ± 0.48	4.39 ± 0.40	4.69 ± 0.51	4.62 ± 0.32

**Table 4:** DTS (after 1 and 7 days of setting) of MTA-like cement prepared with 20 and 40 wt.% of various calcined BTO powders.

DTS (MPa)		PC	600 °C	800 °C	900 °C	1000 °C	1100 °C
20% BTO	1 Day	1.92 ± 0.22	1.51 ± 0.53	1.44 ± 0.17	1.50 ± 0.18	2.43 ± 0.29	1.73 ± 0.18
	7 Days	2.48 ± 0.22	2.32 ± 0.42	2.37 ± 0.58	2.16 ± 0.28	2.46 ± 0.55	2.08 ± 0.31
40% BTO	1 Day	1.92 ± 0.22	0.77 ± 0.26	1.22 ± 0.22	1.68 ± 0.20	2.86 ± 0.33	2.55 ± 0.44
	7 Days	2.48 ± 0.22	0.58 ± 0.27	1.77 ± 0.42	1.82 ± 0.65	3.13 ± 0.70	2.63 ± 0.60

**Table 5:** Biocompatibility evaluation of MTA-like cement prepared using 40 wt.% 1000 °C-calcined BTO powders. MTA-like cement prepared using Portland cement without BTO addition was used for comparison.

Cell Line	MG63		L929	
Cell Viability (%)	PC	40% BTO	PC	40% BTO
1/10,000	95.64 ± 2.45	97.27 ± 6.94	96.02 ± 2.61	95.64 ± 2.45
1/1000	97.43 ± 1.06	97.77 ± 1.62	97.10 ± 3.43	98.47 ± 2.20
1/100	96.02 ± 2.61	100.71 ± 2.31	100.72 ± 2.3	97.27 ± 6.94

## Data Availability

Data are contained within the article.
